# Copepod Mortality due to Short‐Term Exposure to Natural Ultraviolet Radiation at Subtropical Latitudes

**DOI:** 10.1002/ece3.71701

**Published:** 2025-06-27

**Authors:** Samuel Hylander, Jeremias Nhaca, Ilário Timba, Marc M. Hauber, David V. P. Conway, Salomão Bandeira

**Affiliations:** ^1^ Department of Biology and Environmental Science, Centre for Ecology and Evolution in Microbial Model Systems (EEMiS) Linnaeus University Kalmar Sweden; ^2^ Inhaca Marine Biology Station Universidade Eduardo Mondlane Maputo Mozambique; ^3^ Marine Biological Association of the United Kingdom Plymouth UK; ^4^ Department of Biological Sciences Universidade Eduardo Mondlane Maputo Mozambique

**Keywords:** mortality, UV, zooplankton

## Abstract

Zooplankton, particularly copepods, are key components in aquatic food webs. However, the effects of ultraviolet (UV) radiation on copepods in marine systems, especially at tropical and subtropical latitudes, are not well understood. Incubations in UV and non‐UV treatments during outdoor solar experiments at a subtropical latitude where copepods dominated the zooplankton community demonstrated that UV exposure led to 40%–50% higher mortality than in non‐UV treatments after 4 h of exposure. In outdoor plankton migration tower experiments, most copepods avoided surface waters regardless of radiation treatment. While adaptations to avoid UV damage, such as the accumulation of photoprotective compounds, were observed in copepods, they were insufficient to fully mitigate UV‐induced harm. Thus, surface avoidance is likely the primary adaptation employed by copepods and other zooplankton to evade UV exposure. This study expands upon existing UV research, which has largely focused on high‐latitude and high‐altitude ecosystems, suggesting that UV is a major environmental threat factor for low‐latitude zooplankton. Hence, projected future climate‐change related or geoengineering‐driven increases in UV levels in subtropical and tropical systems may lead to higher mortality rates in zooplankton populations.

## Graphical abstract

Zooplankton are key nodes in the food web. We demonstrate that approximately 40%–50% of the copepods died following a few hours of exposure to outdoor ultraviolet radiation (UV). The defence mechanisms of these organisms were insufficient to mitigate this environmental threat. The broader context of this research is the need for a better understanding of UV as an environmental variable, especially given that climate change and proposed geoengineering methods to manipulate the climate are projected to alter UV exposure levels in the future.

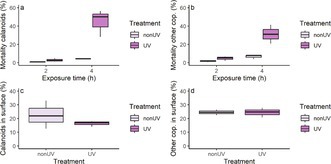

## 1 Introduction

One of the major successes in environmental management has been the implementation of the Montreal Protocol and the subsequent protection of the ozone layer (Barnes et al. 2019). The ozone layer protects Earth from short‐wavelength UV, which is particularly harmful to organismal health and survival, primarily due to DNA damage (Barnes et al. 2019). Emissions of ozone‐depleting substances have decreased, and the ozone layer is expected to recover over the course of this century, reducing the risk of future excess UV radiation reaching Earth (Barnes et al. 2019; WMO 2018).

Ozone depletion has been most pronounced in high latitudes, and as a result, much of the research on UV radiation has focused on these regions (Barnes et al. 2019). Studies have demonstrated that aquatic organisms, including copepods, the focus of this study, are sensitive to UV radiation (Neale et al. 2023) and if exposed show increased mortality and DNA damage (Williamson et al. 2001, 1994). However, the magnitude of the UV effects are influenced by factors such as ice cover, water transparency, and vertical mixing, all of which can modulate UV exposure (Neale et al. 2023) and copepods in these high‐latitude systems also possess a variety of defence mechanisms to mitigate UV damage (Hansson and Hylander 2009; Hylander 2020; Rautio and Tartarotti 2010). Copepods in general play a critical role in aquatic food webs, as they are the primary consumers of phytoplankton and a key prey item for juvenile fish. Reduction in copepod abundance could therefore have vast consequences for fish populations and human food security.

While there has been progress in protecting the ozone layer at high latitudes, recent climate models suggest that global warming could alter future UV exposure in new ways. For example, under the RCP6.0 climate change scenario, there could be a 15% increase in DNA‐damaging UV radiation at subtropical and tropical latitudes (Meul et al. 2016). There is also a significant risk of increasing UV exposure if geoengineering is implemented at large scale, for example by injecting sulphate aerosols into the stratosphere to reduce global temperatures (Barnes et al. 2019; Crutzen 2006). This change in UV exposure could be particularly problematic in tropical and subtropical regions, as organisms in these regions are adapted to a relatively stable UV environment. This underscores the importance of gaining a deeper understanding of how UV radiation affects organisms in low‐latitude systems.

Although there is a wealth of information from the high‐latitude systems, surprisingly little is known about UV sensitivity and defense strategies in zooplankton from tropical and subtropical regions. There are only a few reports suggesting that tropical zooplankton accumulate photoprotective compounds (Fileman et al. 2017; Lee et al. 2019; Rahlff et al. 2018), and they appear to be sensitive to general solar exposure, but these previous studies did not discriminate between UV and non‐UV effects (Al‐Aidaroos et al. 2014). Therefore, we conducted a study on copepod UV sensitivity in a subtropical system, our hypothesis being that subtropical copepods are sensitive to UV exposure, but they mitigate the harmful effects by migrating to deeper waters and by possessing photoprotective compounds.

## 2 Methods

All experiments and samplings were conducted in November 2018 at the Inhaca Marine Biology Station, Mozambique (26°02′19.4″ S, 32°54′07.3″ E), located at the boundary between tropical and temperate climatic zones. The copepod community at the site was sampled via vertical net hauls from a depth of 10 m to the surface using a plankton net (50 cm diameter, 200‐μm mesh size). Field identifications were made using a Nikon field microscope (20×), with some samples preserved in ethanol for later, more detailed taxonomic identification (Conway et al. 2003). Zooplankton in this area include a variety of taxa (Conway et al. 2003) but copepods are main components and the focus of this study. The copepod community was dominated by calanoids but also included notable numbers of harpacticoids and cyclopoids. Juvenile individuals predominated, making precise species identification challenging, although the following species and genera were identified with certainty: *Acrocalanus* spp., *Euterpina acutifrons* , *Oncaea* spp., *Microsetella norvegica* , *Temora* spp., *Temora turbinata* , *Corycaeus* spp., *Calocalanus* spp., and *Oithona* spp.

### 2.1 Mortality

Mortality in response to UV exposure was assessed outdoors in plastic containers (17 cm diameter, 10 cm depth), submerged in a 3 × 2 m pool (0.3 m depth; Figure S1). The pool was located near the shoreline, allowing daily tidal flushing with seawater to maintain the temperature within its natural range. Copepods were exposed to UV radiation on two consecutive days (13–14 November, not the same day for logistical reasons): for 2 h (12:20–14:20) and 4 h (12:00–16:00), under clear skies with occasional overcast. For the non‐UV treatment (*n* = 3), containers were shielded with Plexiglas that blocked radiation below 370 nm (Röhm GS 233). UV treatment replicates (*n* = 3) were covered with UV‐transparent Plexiglas allowing all UV wavelengths to enter (Röhm GS 2458). Transmission of photosynthetically active radiation was similar between the two types of Plexiglas, and the full transmission spectra are available in Hansson et al. (2007). Each container was filled with 1.25 L of water (pre‐filtered at 15 μm) that allowed zooplankton to utilize any phytoplankton that were in the water, and no other food items were added. Each replicate was then stocked with 360 ± 11 and 198 ± 5 copepods per replicate for the 2‐h and 4‐h exposures, respectively. Hence, all replicates contained a mixture of different zooplankton taxa, and copepods were not specifically picked out to avoid harm and elevated mortality due to excessive handling. Upon termination of the exposure, each replicate was concentrated to 100 mL and stored in a glass bottle in the shade until counted under a stereomicroscope (Nikon field microscope, 20×). Mortality was determined by a continued lack of movement. Copepods were categorized as either calanoid copepods (adults and copepodites) or other copepod taxa (all other groups without separating taxa (such as cyclopoids, harpacticoids; and all life‐stages (adults, copepodites and nauplii))). Temperature remained consistent between UV and non‐UV treatments after incubation (2‐h exposure: UV = 30.6 ± 0.10°C, non‐UV = 30.6 ± 0.06°C; 4‐h exposure: UV = 30.6 ± 0.06°C, non‐UV = 30.6 ± 0.10°C; average ± SD).

### 2.2 Migration

Surface avoidance behaviour in copepods was quantified using two 99 cm long, UV‐transparent, cylindrical migration towers (9.5 cm diameter; Plexiglas XT 29070 (0A070)), partially submerged in the same pool to maintain stable temperature. These towers transmit 80% of UVA and 50% of UVB radiation on average (Buchner et al. 2013). Zooplankton were placed in the upper opening of the cylinders and incubated for 2 h during midday (solar noon) under either full solar radiation or a non‐UV treatment, using the same zooplankton stock solution as above. Following incubation, the cylinders were separated into two equal compartments using an internal partition mechanism, allowing separate collection of the zooplankton in the top and the bottom. The non‐UV treatment was achieved by wrapping one cylinder in UV filter film (UV CL SR HPR), which blocked all radiation below 400 nm and transmitted less than 0.1% of UV wavelengths (LLumar 2010). The top of the UV treatment cylinder was covered with UV‐transparent Plexiglas, while the non‐UV cylinder was covered with Plexiglas that blocked UV radiation (see above). This experiment was repeated over three consecutive days (13–15 November, not the same day for logistical reasons), which are treated as replicates in subsequent analyses and figures. All days had clear skies with some overcast. Copepods in the samples were counted under a microscope (20× magnification) and categorized as either calanoids or other copepods. Temperature remained consistent between UV and non‐UV treatments after cylinder incubations (UV = 30.6 ± 0.17°C, non‐UV = 30.6 ± 0.15°C; average ± SD).

### 2.3 UV Environment and Photoprotective Compounds

UVA radiation was measured in depth gradients over three consecutive days at the sampling location using UVA sensor SUL 240, connected to a IL 1400A logger (International light, Newburyport, Massachusetts, USA). To assess the potential presence of mycosporine‐like amino acids (MAAs) in the copepod community, two common and easily distinguishable taxa—*Temora* spp. (calanoid) and *Euterpina acutifrons* (harpacticoid)—were selected. In three replicates per taxon, 10–23 *Temora* individuals and 60 *Euterpina* individuals were sampled and MAAs were extracted in aqueous methanol according to (Tartarotti and Sommaruga 2002). In short, zooplankton were homogenised with a sterile glass rod and then extracted twice in 2 mL methanol (45% alcohol) in a 45°C water bath. Absorbance was quantified at 3 nm intervals between 325 and 400 nm to detect the potential for MAAs in copepod tissues using samples that had been pre‐filtered (Whatman GF/C) to remove any copepod debris. MAAs were calculated as the absorbance per dry weight. Additionally, three mixed copepod population samples were extracted in ethanol (6 h in room temperature) according to (Hylander et al. 2009) to quantify carotenoids, including astaxanthin. All data was recorded from instruments and typed into Excel and statistics and figures were prepared using RStudio (version 2021.09.0). Both mortality and migration were calculated as percentages of the total population that either died in the mortality experiment or remained in the upper part of the migration towers in the migration experiment. Differences between treatments were analysed with independent samples *t*‐test (assuming unequal variances) after log‐transforming the data. In one case (calanoids mortality after 4 h), all replicates had the same number of animals dying (non‐UV treatment) leading to zero variance among replicates. In this case a one sample *t*‐test was used to compare the value in the non‐UV treatment to the results for the UV‐treatment replicates.

## 3 Results

### 3.1 Mortality of Copepods in Response to UV Exposure

**FIGURE 1:**
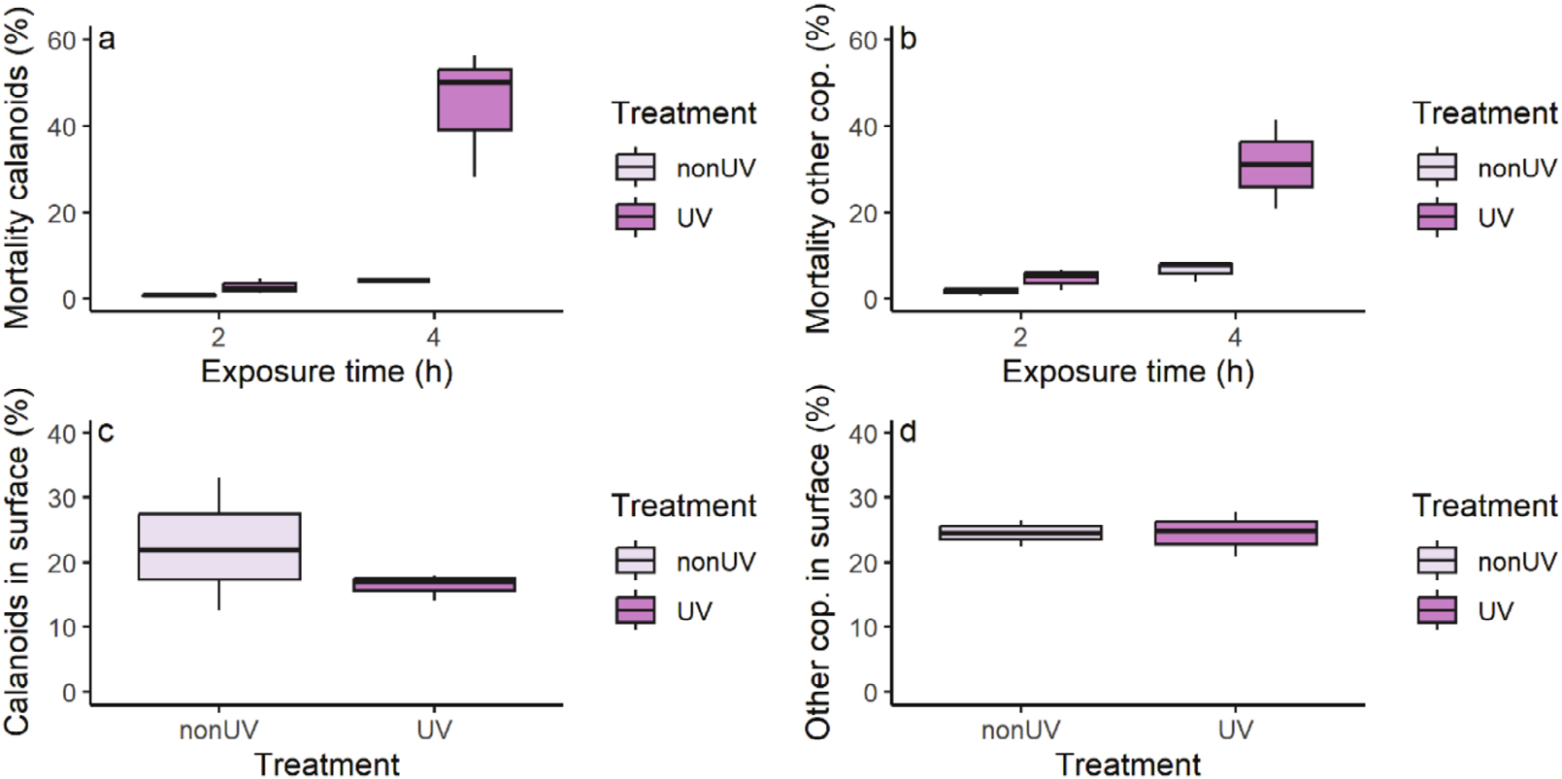
Mortality of calanoid copepods (a) and all other copepods (b) during two‐ and four‐hour incubations in surface waters under UV and non‐UV treatments. Subfigures (c) and (d) show the percentage of calanoid copepods and all other copepods in the upper half of cylindrical migration towers compared to those in the lower half under UV and non‐UV treatments.

The zooplankton community was primarily composed of calanoid copepods (adults and copepodites), which numerically accounted for 40%–60% of the total abundance, with smaller contributions from other copepod taxa (all other copepod specimens). Mortality in the calanoid copepod community was 2.8% ± 1.7% (mean ± SD) after 2 h of UV exposure, increasing to 44.8% ± 14.8% (mean ± SD) after 4 h (Figure 1a). Background mortality was 1.2% and 4.1% after and 4 h of exposure, respectively. Similar patterns of mortality were observed in the other copepods (Figure 1b). Calanoid copepods exhibited significantly higher mortality in the UV treatments compared to the non‐UV treatments, both after 2 h (*t* = −2.9, *p* = 0.053, df = 3.5; marginally significant) and 4 h of exposure (*t* = 10.9, *p* = 0.008, df = 2). Similarly, all other copepod groups showed higher mortality in the UV treatment compared to the non‐UV treatment after 4 h (*t* = −4.5, *p* = 0.014, df = 3.6), but not after 2 h (*t* = −1.8, *p* = 0.15, df = 3.9).

### 3.2 Migration of Copepods in Response to UV Exposure

When calanoid copepods were allowed to choose their depth distribution in cylindrical migration towers under different UV regimes, 16.3% ± 2.0% (mean ± SD) remained in the upper half of the towers under UV treatment, compared to 22.5% ± 10.2% (mean ± SD) under non‐UV treatment (Figure 1c). Similar proportions of other copepod species remained in the upper half of the towers. Overall, the majority of copepods avoided the upper portion of towers, and there was no significant difference in numbers between UV and non‐UV treatments for either calanoid copepods or other copepod species (calanoids: *t* = 0.9, *p* = 0.47, df = 2.3; other copepods: *t* = 0.94, *p* = 0.43, df = 2.5).

### 3.3 UV Environment In Situ and Potential for Photoprotection

**FIGURE 2:**
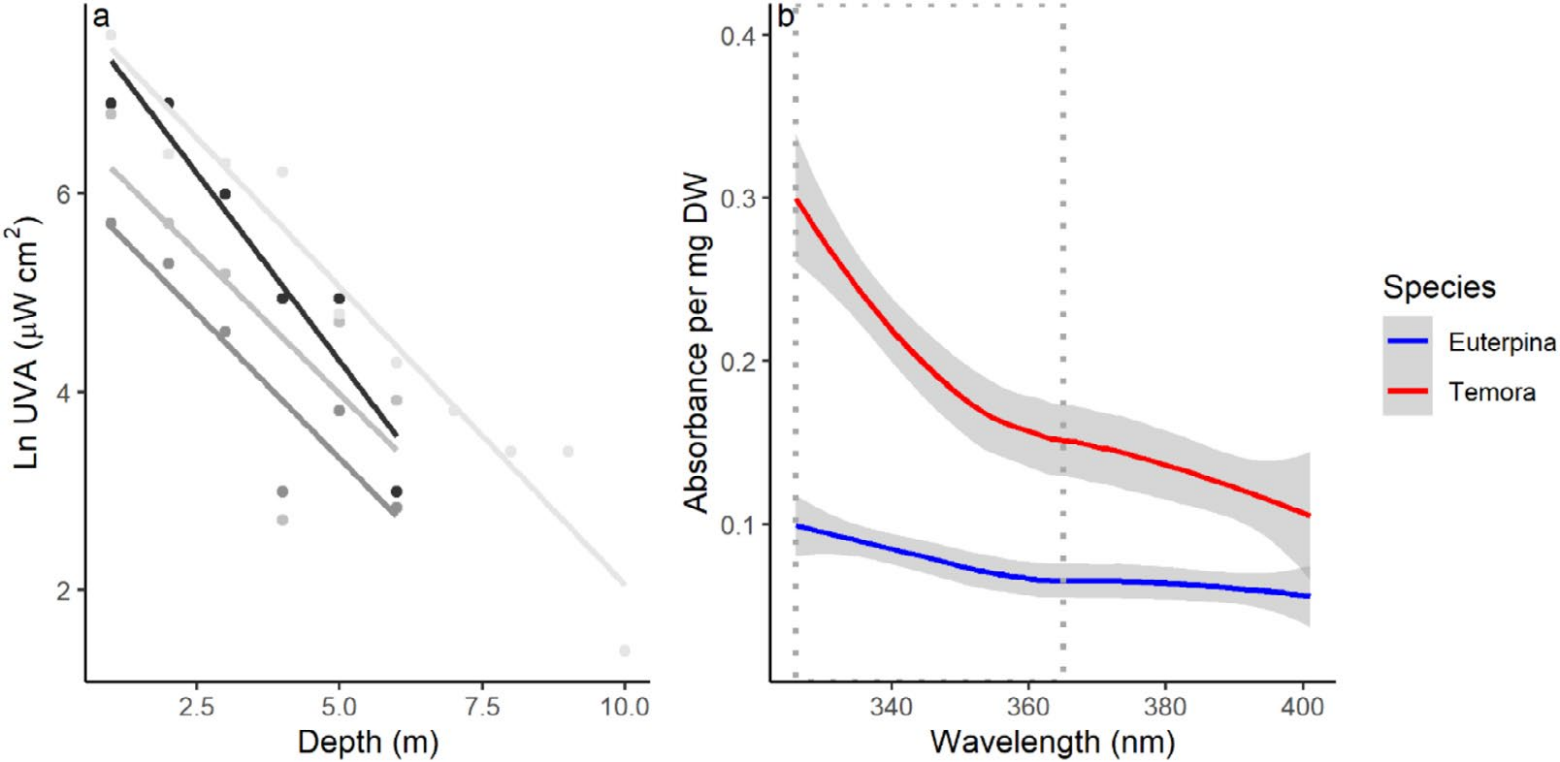
UVA intensity at different depths (ln‐transformed) measured four times (different lines) over three consecutive days at the sampling location (a) and absorbance of methanol extracts (b) from two common copepod species ( *E. acutifrons* and *Temora* spp.) sampled at the same site. Grey dotted box indicate the region where most MAAs have their absorption maximum in the quantified wavelength region.

UVA intensity decreased with depth at the sampling site (Figure 2), with an average K_dUVA_ of 0.63 ± 0.08 (mean ± SD) and a 1% attenuation depth of UVA at 7.4 ± 0.8 m (mean ± SD). Therefore, zooplankton at this coastal site are exposed to UV within the first 7–8 m of the water column. Two common taxa, the calanoid *Temora* spp. and the harpacticoid *Euterpina acutifrons* , were extracted in methanol (see methods) to detect potential mycosporine‐like amino acids (MAAs). Absorption in the copepod extracts was higher at the absorption maxima of common MAAs (e.g., porphyra and shinorine, absorption maximum 325–340 nm) than at longer wavelengths (360–400 nm), where MAAs do not exhibit significant absorbance. *Temora* spp. showed higher absorbance per dry weight in the MAA absorption range compared to *E. acutifrons* . Additionally, three samples with a mixed copepod population were extracted in ethanol (see methods) to detect potential astaxanthin. However, no absorbance was detected at the maximum absorption peak of astaxanthin (474 nm). Copepods also appeared transparent, lacking visible pigments. These observations suggest that astaxanthin pigmentation is low in this copepod community, although some species may possess MAAs.

## 4 Discussion

We demonstrate that just a few hours of natural solar UV exposure results in mortality rates of 40%–50% compared to non‐UV treatments. Defence mechanisms in zooplankton were insufficient to mitigate this environmental threat, which likely forces them to avoid surface waters in these systems. This work expands the current understanding by investigating UV effects on zooplankton in a coastal marine subtropical system, whereas most other studies have focused on high‐latitude and high‐altitude environments, predominantly in lakes (Hansson and Hylander 2009; Hylander 2020; Rautio and Tartarotti 2010). This study also complements the literature by being one of the few experiments conducted in natural radiation conditions, as opposed to the more common laboratory studies (Neale et al. 2023). The benefit of using outdoor experiments is that the natural solar spectrum is utilized along with naturally occurring intensities of radiation. Laboratory‐based experiments can be valuable in order to assess UV effects under controlled conditions, for example when quantifying the spectral sensitivity of a species, or studying the mechanisms for UV defence (Neale et al. 2023, 2025; Madronich et al. 2024). However, natural solar radiation is challenging to mimic in the laboratory, and it is crucial that laboratory‐based experiments use exposures that are relevant both in terms of spectral composition and intensity, as well as utilizing outdoor experiments with natural radiation to verify the absolute effects of UV exposure in the ecosystem (Neale et al. 2023, 2025; Madronich et al. 2024). This study demonstrates that natural radiation is enough to cause significant mortality in low‐latitude zooplankton.

Previous estimates of mortality in tropical and subtropical zooplankton are limited. Al‐Aidaroos et al. (2014) showed that increasing solar radiation elevates mortality in several copepod species. For example, the average mortality rates among different zooplankton species were in the order of five times higher in the full radiation treatments compared to dark controls, but without distinguishing between different wavelengths of the solar spectrum (Al‐Aidaroos et al. 2014). We did not discriminate between UVA and UVB effects, but UV‐induced mortality in zooplankton has been shown to be primarily caused by the UVB proportion of the spectrum (Neale et al. 2023; Williamson et al. 2001). Cohort studies have furthermore demonstrated that UV exposure leads to significant fitness loss in zooplankton (Hylander et al. 2014). Hence, tropical and subtropical zooplankton face a significant environmental threat if exposed to UV radiation. Background mortality was low in the non‐UV treatments, and temperatures were similar between treatments, suggesting that UV led to the observed mortality. However, we cannot exclude the possibility that an unknown factor correlating with UV exposure affected the results.

Zooplankton have several adaptations to mitigate UV damage, including the accumulation of mycosporine‐like amino acids (MAAs), pigmentation, internal antioxidant systems, vertical migration, and repair mechanisms (Hansson and Hylander 2009; Hylander 2020; Rautio and Tartarotti 2010; Vilgrain et al. 2023). Here, despite these potential defenses, zooplankton could not fully counteract the negative consequences of solar UV exposure, although we did observe absorbance in copepod tissues consistent with the presence of MAAs. Pigmentation was low, suggesting that astaxanthin is scarce in this zooplankton community. Astaxanthin is known to be beneficial to reduce oxidative stress (e.g., due to UV‐exposure) but at the cost of higher visibility and predation mortality (Vilgrain et al. 2023; Brüsin et al. 2016). Most of the previous knowledge on UV defense mechanisms has been developed in high latitude and altitude lake systems (Hansson and Hylander 2009; Hylander 2020) and it is not well known whether UV response adaptations vary consistently between marine and freshwater zooplankton and along latitudinal gradients. Marine systems are often deeper, allowing for a UV depth refuge, but can have shallow mixed layers, whereas many lake systems can be shallow but also sometimes rich in colored dissolved organic matter reducing the UV transparency (Neale et al. 2023). It has been shown that freshwater copepods are more pigmented compared to marine copepods, and it has been speculated that this might be due to higher UV exposure in lakes, but pigmentation can also have several other benefits to the organisms (Hylander et al. 2014; Vilgrain et al. 2023). In terms of MAAs, they tend to be similar between freshwater and marine systems, although comparisons are scarce (Hansson and Hylander 2009; Hylander 2020), and it has been suggested that copepods in temperate marine systems are richer in MAAs compared to tropical copepods (Fileman et al. 2017).

Cladocerans have been shown to avoid UV with downward migration, whereas copepods show a variable response with weak or no avoidance (Hansson et al. 2007; Hylander et al. 2009; Aarseth and Schram 1999; Boeing et al. 2004; Speekmann et al. 2000). In our study, a majority of the copepods incubated in migration towers avoided the surface, regardless of UV exposure. This implies that this behavior is not directly triggered by the UV proportion in the solar spectrum. Avoidance of surface waters has been regarded as an adaptation to avoid predation from fish, and this behavior would also reduce exposure to UV radiation (Hansson and Hylander 2009). Temperature was similar between treatments, suggesting that some other factors triggered the zooplankton to avoid the surface regardless of UV treatment.

We demonstrate that in subtropical systems, where UV irradiance is often much higher than in high‐latitude systems, natural solar UV exposure for just a few hours is sufficient to cause significant mortality in copepods and potentially other zooplankton. Our results also suggest that UV radiation serves as a significant environmental stressor that forces copepods to avoid surface waters, reducing their ability to graze and serve as prey items for higher trophic levels, such as fish. Given the projected changes in incident UV radiation and water transparency in subtropical and tropical systems, UV‐induced mortality may increase in these regions in the future. Hence, a continued management to protect the ecosystems on Earth from excessive UV exposure is essential.

## Author Contributions

**Samuel Hylander:** conceptualization (lead), funding acquisition (lead), investigation (lead), methodology (lead), project administration (lead), supervision (lead), visualization (lead), writing – original draft (lead), writing – review and editing (lead). **Jeremias Nhaca:** investigation (supporting), methodology (supporting), writing – review and editing (equal). **Ilário Timba:** investigation (supporting), methodology (supporting), writing – review and editing (equal). **Marc M. Hauber:** investigation (supporting), methodology (supporting), visualization (supporting), writing – review and editing (equal). **David V. P. Conway:** investigation (supporting), methodology (supporting), writing – review and editing (equal). **Salomão Bandeira:** conceptualization (supporting), funding acquisition (supporting), investigation (equal), methodology (equal), project administration (equal), writing – review and editing (equal).

## Conflicts of Interest

The authors declare no conflicts of interest.

## Supporting information


Data S1.

Figure S1.

## Data Availability

All raw data is available in Data S1, and a description of the raw data is available in the readme sheet in the same file.
