# Supplementary material for: Copepod Mortality due to Short‐Term Exposure to Natural Ultraviolet Radiation at Subtropical Latitudes
Source: Ecol Evol. 2025 Jun 27;15(7):e71701. doi: 10.1002/ece3.71701 (PMC12203230; doi:10.1002/ece3.71701)
Supplement: Supplementary file 2 — Figure S1. [file ECE3-15-e71701-s002.docx]

Supporting information: Copepod mortality due to short-term exposure to natural ultraviolet radiation at subtropical latitudes

Samuel Hylander^1^*, Jeremias Nhaca^2^, Ilário Timba^2^, Marc M. Hauber^1^, David V.P. Conway^3^, Salomão Bandeira^4^

^1^Department of Biology and Environmental Science, Centre for Ecology and Evolution in Microbial Model Systems (EEMiS), Linnaeus University, Kalmar, Sweden.

^2^ Inhaca Marine Biology Station, Universidade Eduardo Mondlane, Inhaca Island, Mozambique.

^3^ Marine Biological Association of the United Kingdom, The Laboratory, Citadel Hill, Plymouth PL1 2PB, UK.

^4^Department of Biological Sciences, Universidade Eduardo Mondlane, Maputo, Mozambique

* Corresponding author: samuel.hylander@lnu.se, ORCID: 0000-0002-3740-5998

**Description of raw data**

All raw data used in this manuscript is available in the file S1 raw data Hylander. Sheet 1 is mortality data, sheet 2 migration data, sheet 3 MAAs and sheet 4 attenuation of UV radiation and units are available in the raw data file. There are no licences or restrictions placed on accessing and using the dataset.

Mortality and migration estimates were performed in experimental vessels in fig S1.


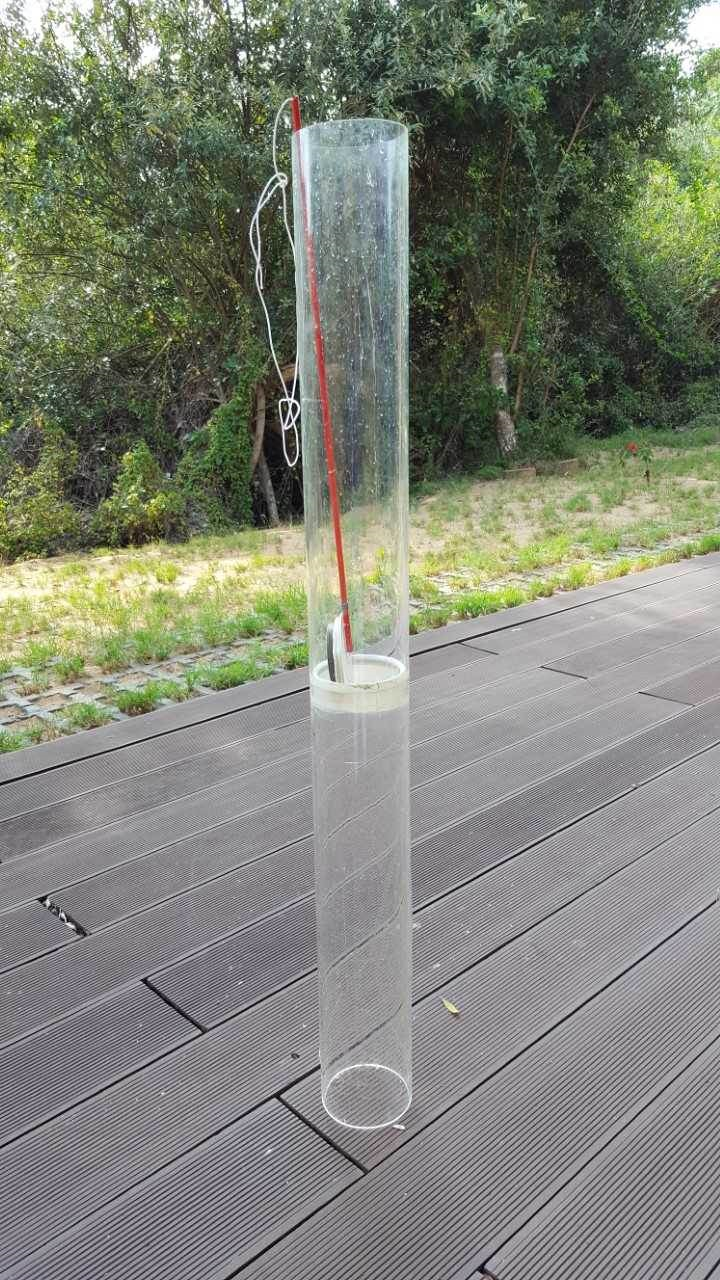

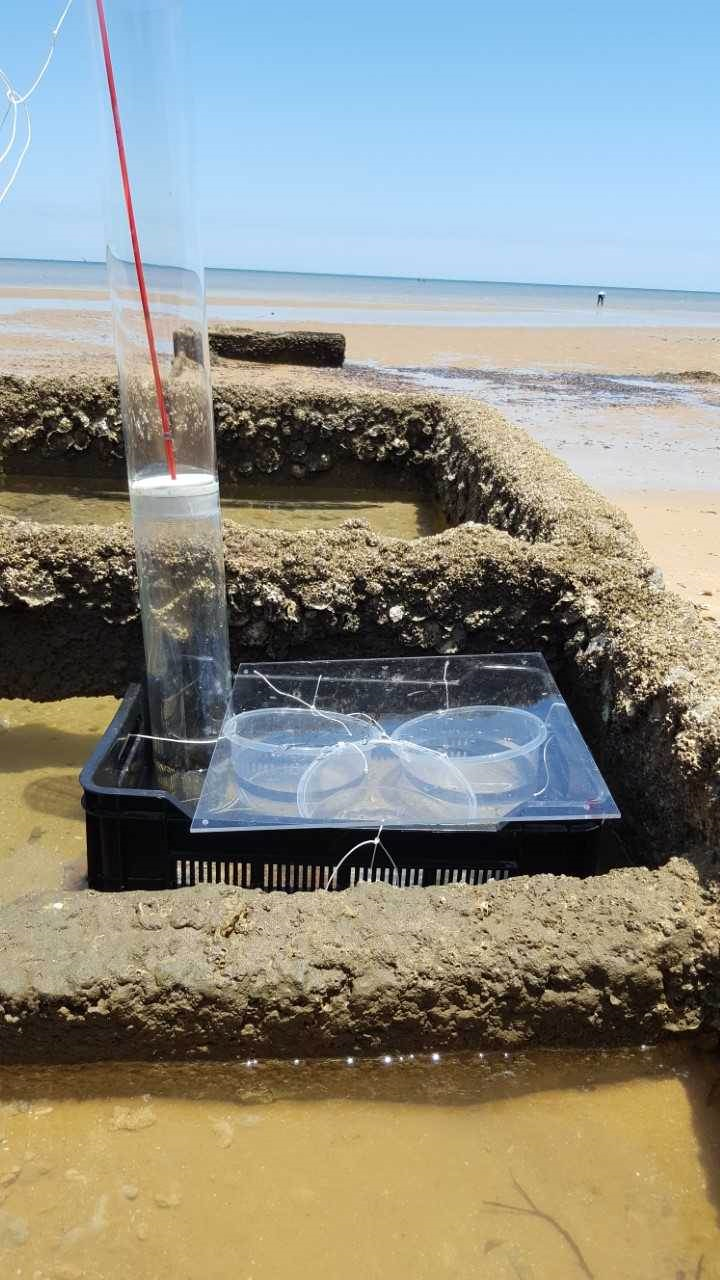


Fig. S1. Migration estimates were performed in cylinders that could be closed after a certain amount of time using the red rod (left). Experimental vessels used in the mortality estimates were placed under different types of screens to regulate UV exposure (right; Photos: Samuel Hylander).
